# Supplementary material for: Expression and Biological Functions of miRNAs in Chronic Pain: A Review on Human Studies
Source: Int J Mol Sci. 2022 May 27;23(11):6016. doi: 10.3390/ijms23116016 (PMC9181121; doi:10.3390/ijms23116016)
Supplement: Supplementary file 1 [file ijms-23-06016-s001.zip › ijms-1694017-supplementary.pdf]

Supplementary Materials

Table S1. Target genes of miRNAs in CPP and CSP predicted by TargetScanVert, miRDB and miRWalk (the target names are arranged in alphabetical order).

ID	Expression	Target
hsa-let-7a-5p	↓	A1CF
		AAK1
		ABCC5
		ABHD17C
		ABL2
		ACER2
		ACSL6
		ACVR1C
		ADAMTS15
		ADIPOR2
		ADRB1
		ADRB2
		ADRB3
		AEN
		AFF2
		AGAP1
		AHCTF1
		AIFM1
		AKAP6
		AMER3
		AMMECR1L
		AMOT
		AMT
		ANKFY1
		ANKRD46
		ANKRD52
		AP1S1
		APBA1
		ARG2
		ARHGAP28
		ARHGEF15
		ARHGEF38
		ARID3A
		ARID3B
		ARL4D
		ARMC8
		ARPP19
		ASAP1
		ATAD2B
		ATP2A2
		ATP2B4
		ATP6V1C1
		ATP8B4
		B3GAT3
		B3GNT7
		BACH1

	BBX
	BCAP29
	BCAT1
	BCL2L1
	BCL7A
	BEND4
	BIN3
	BRWD3
	BSN
	BTBD3
	BTBD9
	BZW1
	BZW2
	C14orf28
	C15orf39
	C19orf47
	C20orf194
	C5orf51
	CACNA1E
	CACNA1I
	CACNB4
	CADM2
	CALU
	CAPN3
	CASKIN1
	CBFA2T3
	CBX5
	CCND1
	CCND2
	CCNJ
	CCNT2
	CCNY
	CCR7
	CDC25A
	CDC34
	CDKN1A
	CDV3
	CEP164
	CERCAM
	CFL2
	CGNL1
	CHD4
	CHIC1
	CHRD
	CHSY3
	CLASP2
	CLCN5
	CLDN12
	CLOCK
	CLP1
	CNOT6L
	CNTRL

	COIL
	COL14A1
	COL1A1
	COL1A2
	COL27A1
	COL4A1
	COL4A2
	COL4A6
	COL5A2
	COL9A1
	CPA4
	CPEB1
	CPEB2
	CPED1
	CPM
	CPSF4
	CRB2
	CRK
	CRTAP
	CRY2
	CSRNP3
	CYB561D1
	DAGLA
	DAPK1
	DARS2
	DCAF15
	DCUN1D3
	DDI2
	DDX19A
	DDX19B
	DHX57
	DICER1
	DKK3
	DLC1
	DLGAP4
	DLST
	DMD
	DOCK3
	DPH3
	DPP3
	DST
	DTX4
	DUSP16
	DUSP22
	DUSP7
	DVL3
	DYRK1A
	DYRK2
	E2F2
	E2F5
	E2F6
	EDEM3

	EDN1
	EEA1
	EEF2K
	EGLN2
	EGR3
	EIF2S2
	EIF4G2
	ELF4
	ELK4
	ELOVL4
	ENTPD7
	EOGT
	EPHA3
	EPHA4
	ERCC4
	ERCC6
	ERGIC1
	ESPL1
	ETNK1
	ETNK2
	FAM104A
	FAM118A
	FAM135A
	FAM189A1
	FAM214B
	FASLG
	FAXC
	FBXO30
	FBXO32
	FBXO45
	FGD6
	FGF11
	FIGN
	FKRP
	FNDC3A
	FNDC3B
	FNIP1
	FNIP2
	FOXP2
	FRAS1
	FREM2
	FRMD4B
	FRMD5
	FRS2
	FZD3
	FZD4
	GAB2
	GABPA
	GABRA6
	GALNT1
	GALNT15
	GALNT4

	GAN
	GAS7
	GCNT4
	GDAP2
	GDPD1
	GHR
	GIPC1
	GJC1
	GLRX
	GNG5
	GNPTAB
	GOLGA4
	GOLT1B
	GPATCH3
	GPR132
	GPR156
	GPR26
	GRAMD1B
	GRID2IP
	GRIN3A
	GRPEL2
	GTF2I
	GYG2
	HAS2
	HDLBP
	HDX
	HECTD2
	HELZ
	HIC2
	HIF1AN
	HIF3A
	HIP1
	HK2
	HMGA1
	HMGA2
	HOOK1
	HOXA1
	HOXA9
	HOXD1
	HS2ST1
	HTR1E
	ICMT
	IGDCC3
	IGDCC4
	IGF1
	IGF1R
	IGF2BP1
	IKZF2
	IL10
	IL13
	IMPG2
	INPP5A

	INSR
	INTS2
	IRS2
	ITGB3
	ITGB8
	KATNAL1
	KATNBL1
	KCNC1
	KCNC2
	KCNJ11
	KCNJ3
	KCNQ4
	KCTD16
	KCTD21
	KHNYN
	KIAA0930
	KIAA1549
	KIAA1958
	KIF21B
	KLF8
	KLF9
	KLHDC8B
	KLHL31
	KLHL6
	KLK10
	KMT2D
	KPNA1
	KREMEN1
	L2HGDH
	LBH
	LBR
	LCORL
	LDB1
	LEPROTL1
	LIMD2
	LIMK2
	LIN28A
	LIN28B
	LINGO1
	LIPH
	LIPT2
	LMLN
	LMX1A
	LOXL4
	LPGAT1
	LRFN4
	LRIG2
	LRIG3
	LRRC8B
	LSM11
	LYVE1
	MAP3K1

	MAP3K2
	MAP3K3
	MAP3K9
	MAP4K3
	MAP4K4
	MAPK8
	MARS2
	MBD2
	MBTPS2
	MDFI
	MDM4
	MED28
	MED6
	MEF2C
	MEIS2
	MEX3A
	MGA
	MGAT4A
	MGLL
	MIB1
	MIEF1
	MLLT10
	MMP11
	MON2
	MRS2
	MSI2
	MTDH
	MTUS1
	MXD1
	MYCBP
	MYCN
	MYO1F
	MYO5B
	MYRIP
	NAA30
	NAP1L1
	NAT8L
	NDST2
	NEK3
	NGF
	NIPA1
	NIPAL4
	NKD1
	NKIRAS2
	NME4
	NME6
	NPEPL1
	NR6A1
	NRAS
	NRK
	NSD1
	NYNRIN

	OLR1
	ONECUT2
	OSBPL3
	OSMR
	OSTF1
	OTOF
	P2RX1
	P4HA2
	PAG1
	PALD1
	PALM3
	PAPPA
	PARD6B
	PARM1
	PARP8
	PAX3
	PBX1
	PBX2
	PBX3
	PCDH19
	PCDH20
	PCGF3
	PDE12
	PDGFB
	PDP2
	PDPR
	PEG10
	PEX11B
	PGM2L1
	PGRMC1
	PIGA
	PIK3CA
	PIK3IP1
	PITPNM3
	PKN2
	PLAGL2
	PLCXD3
	PLD3
	PLD5
	PLEKHA8
	PLEKHG7
	PLEKHH1
	PLEKHO1
	PLXNA4
	PLXNC1
	PLXND1
	POC1B-GALNT4
	POGLUT1
	POGZ
	POLR2D
	POLR3D
	POTEM

	POU2F1<br>PPARGC1B<br>PPP1R12B<br>PPP1R15B<br>PPP1R16B<br>PPP2R2A<br>PPP3CA<br>PRDM1<br>PRDM5<br>PRKAA2<br>PRLR<br>PRPF38B<br>PRRX1<br>PRSS22<br>PTAFR<br>PTPRD<br>PXDN<br>PXT1<br>RAB11FIP4<br>RAB40C<br>RAB8B<br>RAG1<br>RALB<br>RANBP2<br>RASGRP1<br>RB1<br>RBFOX1<br>RBFOX2<br>RBMS1<br>RBMS2<br>RDH10<br>RDX<br>RFX6<br>RGS16<br>RGS6<br>RNF165<br>RNF170<br>RNF20<br>RNF217<br>RNF38<br>RNF44<br>RORC<br>RPS6KA3<br>RPUSD2<br>RRM2<br>RSPO2<br>RUFY3<br>SALL4<br>SAMD12<br>SBK1<br>SBNO1<br>SCN4B

	SCN5A
	SCYL3
	SDK1
	SEC31B
	SECISBP2L
	SEMA4C
	SEMA4F
	SEMA4G
	SENP2
	SENP5
	SESN3
	SFMBT1
	SH2B3
	SKIDA1
	SKIL
	SLC10A7
	SLC12A9
	SLC16A14
	SLC16A9
	SLC1A4
	SLC20A1
	SLC22A23
	SLC25A24
	SLC30A4
	SLC31A1
	SLC35D2
	SLC38A9
	SLC45A4
	SLC52A3
	SLC5A6
	SLC5A9
	SLC7A14
	SLK
	SMAD2
	SMARCAD1
	SMC1A
	SMCR8
	SMIM13
	SMUG1
	SNAP23
	SNX16
	SNX30
	SNX6
	SOCS1
	SOCS4
	SOCS7
	SOWAHA
	SOX13
	SOX6
	SPATA2
	SPEG
	SPRYD4

	SPTBN4
	SRD5A3
	SREBF2
	SREK1IP1
	SRGAP1
	STARD13
	STARD9
	STEAP3
	STK24
	STK40
	STOX2
	STRBP
	STRN
	STX17
	STX3
	STXBP5
	SUB1
	SULF2
	SURF4
	SYNCRIP
	SYT1
	SYT11
	SYT2
	SYT7
	TAB2
	TAF9B
	TBX5
	TEAD3
	TECPR2
	TENM2
	TET2
	TET3
	TEX261
	TGDS
	TGFBR1
	TGFBR3
	THBS1
	TMC7
	TMED5
	TMEM167A
	TMEM234
	TMEM41A
	TMEM65
	TMOD2
	TMPRSS2
	TMX4
	TNFSF9
	TP53
	TPP1
	TRABD
	TRANK1
	TRHDE

	TRIB1
	TRIB2
	TRIM67
	TRIM71
	TRPM6
	TSC1
	TSC22D2
	TSPAN18
	TSPAN2
	TSPEAR
	TSPYL5
	TTL
	TTLL4
	TUSC2
	TXLNA
	TXLNG
	UBE2G2
	UGCG
	UGGT1
	UHRF2
	USP12
	USP24
	USP32
	USP38
	USP44
	USP49
	UTRN
	VASH2
	VAV3
	VGLL3
	VSNL1
	WARS2
	WDR37
	WIPI2
	WNK3
	WNT9B
	XK
	XKR8
	XRN1
	XYLT1
	YOD1
	YPEL2
	YTHDF3
	YY1
	ZBTB10
	ZBTB39
	ZBTB8B
	ZC3H3
	ZFYVE26
	ZNF10
	ZNF200
	ZNF202

		ZNF275<br>ZNF280B<br>ZNF322<br>ZNF341<br>ZNF516<br>ZNF566<br>ZNF583<br>ZNF641<br>ZNF644<br>ZNF652<br>ZNF689<br>ZNF710<br>ZNF784<br>ZSWIM
hsa-mir-34a-5p	↑	ACVR2B<br>ADAM10<br>AFF4<br>ALCAM<br>AP3S2<br>AREG<br>ASB1<br>ATXN7<br>ATXN7L3<br>BCL2<br>BMP3<br>BTBD11<br>C14orf28<br>C3orf70<br>CACNA1E<br>CACNB3<br>CALCR<br>CAMK4<br>CCDC85A<br>CDC25A<br>CDH4<br>CDIP1<br>CNOT6<br>CNTNAP2<br>CR2<br>CRHR1<br>CTDSP2<br>DCAF7<br>DDX17<br>DLL1<br>E2F3<br>E2F5<br>ELL2<br>ERGIC1<br>FAT3<br>FOXN2<br>FOXN3<br>FRK

		FUT9
		GABRA3
		GAS1
		GMNC
		GNAQ
		GPR85
		HS2ST1
		HSBP1
		IGFBP3
		ISY1-RAB43
		JAG1
		LDHA
		LMAN1
		LRRC55
		MCIDAS
		MET
		MGAT4A
		NPNT
		PDGFRA
		PEG10
		PGRMC2
		PPP3R1
		PREB
		RAB43
		RGS17
		RRAS
		RSPO4
		SCML2
		SCN1A
		SDK2
		SERPINE1
		SERPINF2
		SHANK3
		SHISA7
		SIDT2
		SLCO3A1
		SMIM15
		SNTB2
		SPRN
		TMEM200B
		TNRC18
		TP73
		UNC13C
		VAT1
		VPS4A
hsa-mir-155-5p	↑	AAK1
		ACTA1
		AICDA
		ANKRD6
		ANTXR2
		ATP6V1G1

	ATXN1L
	BACH1
	BAIAP2L1
	BCORL1
	BDNF
	BNC2
	BRD1
	C6orf89
	CACNA2D1
	CARHSP1
	CBL
	CD99L2
	CHD8
	CHD9
	CKAP5
	COPS3
	CREBRF
	CSF2RB
	CSRNP2
	CYB561D1
	DET1
	DMTF1
	DNAJB1
	DUSP14
	DYNC1I1
	EMP2
	FAM135A
	FAM168A
	FBXL17
	FBXO11
	FBXO33
	FUT9
	FXR1
	GABRA1
	GNAS
	GPM6B
	HERC4
	HIF1A
	HIVEP2
	HNRNPA3
	IKBKE
	IL6ST
	INPP5D
	ITK
	KANSL1
	KCNA1
	KDM2A
	KDM5B
	KLF3
	KLHL8
	KPNA1
	KSR1

	LCORL
	LRP1B
	LRRC8B
	MBNL3
	MEF2A
	MEIS1
	MIER3
	MMP16
	MORC3
	MSI2
	MYLK
	NAA50
	NDFIP1
	NFASC
	NFIA
	NR3C2
	NRG3
	OTUB2
	PARD3B
	PELI1
	PIAS1
	PICALM
	PTPRJ
	QKI
	RAB11FIP2
	RAB3B
	RAP1B
	RAPGEF2
	RBAK
	RBMS3
	RCN2
	RCOR1
	RELA
	RFX3
	RGP1
	RORA
	RPS6KA3
	RPS6KA5
	RREB1
	RTKN2
	SALL1
	SATB1
	SEMA5A
	SEPHS1
	SGIP1
	SGK3
	SHOX
	SLC12A6
	SMAD1
	SMAD2
	SMAD5
	SMARCA4

		SOCS1
		SOX10
		SP3
		SPI1
		STXBP5L
		SUFU
		SYVN1
		TBR1
		TBRG1
		TCF4
		TCF7L2
		TENM3
		TERF1
		TLE4
		TM9SF3
		TP53INP1
		TRIM23
		TRIM32
		TRPS1
		TSHZ3
		TSPAN14
		UPP2
		USP48
		USP9X
		VAV3
		WNK3
		XKR4
		YWHAZ
		ZBTB38
		ZIC3
		ZNF236
		ZNF618
		ZNF652
		ZPLD1
		ZSWIM6
hsa-mir-103a-3p	↓	ABHD2
		ABL2
		ACER3
		ACOX1
		ACTG1
		ACVR2B
		ADAMTSL3
		ADCYAP1
		AFF4
		AGFG1
		AK2
		AKIRIN2
		AMMECR1
		AMOT
		ANK1
		ANK3
		ANKFY1

	ANO3
	APBA1
	APPL1
	AQP11
	ARHGAP17
	ARHGAP19
	ARHGAP32
	ARHGAP5
	ARIH1
	ARIH2
	ARL8A
	ARMC1
	ARMC8
	ASH1L
	ASIC1
	ATP13A3
	ATXN1
	ATXN1L
	ATXN7L1
	AVL9
	BACH2
	BAZ2A
	BAZ2B
	BCAT2
	BCL2L13
	BDNF
	BLMH
	BOLA3
	BTG2
	BTLA
	BTRC
	C16orf72
	C2CD5
	CAB39
	CACNA1C
	CACNA2D1
	CALU
	CAMK2G
	CAMKK1
	CAPZA2
	CARM1
	CC2D1B
	CCDC50
	CCDC6
	CCNJ
	CCNYL1
	CDC37L1
	CDK14
	CDK6
	CDK8
	CELSR2
	CEP85L

	CHD1
	CHD2
	CHD6
	CHIC1
	CHRM1
	CHST11
	CLIP1
	CLOCK
	CNNM2
	CNTNAP1
	COBLL1
	CORO2B
	CPEB3
	CRELD1
	CSNK1G2
	CSNK1G3
	CTNND1
	CUL4A
	DCBLD2
	DCP1A
	DCUN1D3
	DESI1
	DICER1
	DKK1
	DLEU7
	DLG5
	DLL1
	DNAJB4
	DNAJC3
	DPP8
	DSC3
	DYNC1LI2
	DYRK2
	EBF1
	EIF4B
	EIF5
	ELK4
	ENPP2
	ENTPD4
	ENTPD7
	ERLIN1
	ESR1
	ETF1
	ETNK1
	EVA1A
	FAF2
	FAM104A
	FAM110C
	FAM117B
	FAM189B
	FAM81A
	FAM98A

	FAT4
	FBXL18
	FBXL20
	FBXW7
	FERMT2
	FEZF1
	FGF2
	FGF7
	FLOT2
	FOXJ2
	FOXP1
	FRYL
	FUCA2
	FURIN
	G3BP2
	GABRG2
	GALNT7
	GATAD2A
	GATAD2B
	GGA3
	GIT2
	GLRA2
	GNPNAT1
	GNS
	GPC6
	GPCPD1
	GPR12
	GPR6
	GRIN2A
	GSKIP
	GSTCD
	HELZ
	HERC2
	HIC2
	HMGA1
	HMGA2
	HTT
	IGSF3
	IHH
	IRF2BP2
	JAKMIP2
	KANK4
	KAT7
	KIF1B
	KIF21A
	KIF23
	KIF3B
	KIF5A
	KIF5C
	KLF4
	KLF7
	KPNA1

	KPNA3
	LARP4B
	LATS2
	LCOR
	LIN28A
	LONRF1
	LRP1B
	LRRC15
	MAPK8
	MBNL1
	MBNL2
	MBNL3
	MCFD2
	MED26
	MEF2D
	MFN2
	MIF4GD
	MMS19
	MSI2
	MTFR1L
	MTMR3
	MTMR4
	MYBL1
	MYH9
	N4BP1
	NAA15
	NAA20
	NAV2
	NDEL1
	NEDD9
	NF1
	NFAT5
	NFIA
	NFIB
	NOS1
	NOVA1
	NPAS2
	NPAS3
	NR1D2
	NR2C2
	NR4A3
	NRIP1
	NSG1
	NUDCD2
	NUP50
	OGT
	OSBPL6
	OTUD4
	PACSIN1
	PAFAH1B2
	PAG1
	PAK3

	PAWR
	PCDH17
	PCGF2
	PDCD10
	PDE3B
	PDE4D
	PDE8B
	PDIK1L
	PDK3
	PDK4
	PDS5A
	PDZD8
	PGRMC2
	PHF20
	PHYHIPL
	PIK3R1
	PITPNA
	PLCB1
	PLEKHA1
	POLDIP2
	PPIP5K2
	PPP2R5C
	PPP6C
	PPP6R2
	PRKAB2
	PRKCE
	PRKG1
	PRPF4B
	PRR14L
	PRRT2
	PSMF1
	PTH
	PTPN4
	PURB
	RAB10
	RAB1B
	RAB30
	RAB40AL
	RAI14
	RAN
	RAP2C
	RASSF5
	RASSF8
	RBAK
	RC3H1
	RCAN1
	REST
	RIMS3
	RNF125
	RNF157
	RNF38
	RORA

	RPUSD1
	RRAGC
	RSPO3
	RUNX1T1
	RYBP
	SALL1
	SALL4
	SATB2
	SBNO1
	SCAF8
	SCAMP5
	SCN1A
	SCN2A
	SDCBP
	SEC22C
	SEMA6A
	SETD5
	SGMS2
	SH3BP4
	SH3BP5
	SIK2
	SIPA1L2
	SIX4
	SLC20A2
	SLC25A37
	SLC26A7
	SLC2A14
	SLC2A3
	SLC35A3
	SLC35G1
	SLC44A1
	SLC45A4
	SLITRK1
	SMARCE1
	SNCG
	SNRK
	SNX12
	SNX18
	SNX3
	SOWAHC
	SOX6
	SPATS2L
	SPRY2
	SPRY3
	SREK1
	SRGAP1
	ST3GAL2
	ST8SIA3
	STAG1
	STRIP1
	STRN
	SUN2

	SUZ12
	SV2C
	SVEP1
	SYDE2
	SYNDIG1
	TAOK3
	TBKBP1
	TDG
	TGIF2
	TGM3
	THOC2
	THSD7A
	THY1
	TJP1
	TLK1
	TM7SF3
	TMEM260
	TMEM47
	TNPO1
	TNRC6B
	TPD52
	TRAF3
	TRIAP1
	TRIM71
	TSPAN5
	TTLL7
	TWF1
	UBE2Q1
	UBE2R2
	UBE4A
	UBL3
	UBR3
	UBR4
	UGGT1
	UHMK1
	UMOD
	UNC80
	UPF2
	USP42
	VAMP8
	VCAN
	VCL
	WNK1
	WNT16
	WWP1
	YAF2
	YTHDC1
	ZBTB10
	ZBTB39
	ZC3H12C
	ZC3H7B
	ZCCHC2

		ZFPM2
		ZHX1
		ZMYM2
		ZNF449
		ZNF711
		ZNRF2
		ZNRF3
hsa-mir-146a-5p	↑	ago-01
		AAK1
		AAK1
		ABCC1
		ABCG1
		ABCG4
		ABL2
		ACKR2
		ACTN4
		ADAM19
		ANKFY1
		ANKRD34A
		APP
		APPL1
		ARMC8
		ATP6V1F
		ATP8B1
		BCORL1
		BEND3
		BICD1
		BMPR1A
		BSN
		C16orf72
		CA10
		CADM4
		CAMK1G
		CAMK2D
		CAPZB
		CBX5
		CCDC6
		CCND2
		CDC42
		CDC42EP3
		CDKN2AIP
		CDS1
		CNTD1
		CNTN2
		CORO2B
		CREBRF
		CSMD2
		CTSE
		CTSK
		DAB2IP
		DCTN3
		DGKB

	DLG2
	DNM1
	DYNLL2
	DYRK1B
	EIF4B
	ELAVL3
	ELOVL4
	EMC10
	ENAH
	EPHB2
	ERBB4
	FAIM2
	FAM168A
	FAM210A
	FAM76A
	FBXL20
	FBXO28
	FBXW2
	FZD1
	GALNT10
	GNA13
	GOSR1
	GPR12
	GPR26
	HIC2
	HNRNPD
	HOOK3
	HP1BP3
	IGF2R
	IKZF4
	IRAK1
	JOSD1
	KCNN3
	KDM2B
	KIF3C
	KLC1
	KLF7
	KLF7
	KLK5
	LCOR
	LCOR
	LIF
	LPCAT3
	LRRC15
	LSAMP
	MAPK14
	MAT2A
	MDM4
	MLLT3
	MMP16
	MSI1
	MYBL1

	MYLK
	MYO18A
	MYT1
	NCOA2
	NFATC3
	NFIX
	NMNAT2
	NOVA1
	NR6A1
	NRSN1
	NSD1
	NUCKS1
	PAK6
	PARS2
	PAX2
	PAX5
	PBX2
	PCDH10
	PCDHA1
	PCDHA10
	PCDHA11
	PCDHA12
	PCDHA13
	PCDHA2
	PCDHA3
	PCDHA4
	PCDHA5
	PCDHA6
	PCDHA7
	PCDHA8
	PCDHA9
	PCDHAC1
	PCDHAC2
	PHLDB1
	PHLPP2
	PITPNA
	PLA2G2F
	PPM1L
	PPP1R11
	PRCP
	PRKAR2A
	PRRT2
	PTCHD1
	PTPRA
	RAB14
	RALGPS2
	RARB
	RERE
	RFX7
	RIC8B
	RIMS2
	RIMS4

	RNF20
	RNF4
	ROBO1
	RSBN1
	RUNX1T1
	SAMD8
	SAR1A
	SCARB1
	SCN3B
	SEC22A
	SEL1L3
	SETD5
	SF1
	SGMS1
	SIX3
	SLC16A2
	SLC24A2
	SLC25A15
	SLC25A53
	SLC38A1
	SLC39A14
	SLC8A1
	SMG7
	SNX30
	SORL1
	SORT1
	SOX13
	SPATA2
	SPATS2
	SPIN3
	SSBP2
	STAC2
	STRBP
	SYNM
	TANC2
	TBC1D20
	TCF12
	TDRKH
	TEAD1
	THRA
	TJP1
	TMEM120B
	TMOD2
	TOM1L2
	TOX3
	TPD52
	TRAF6
	TRIM44
	TRIM5
	TRIM67
	TSN
	UHMK1

		URM1<br>USH1C<br>USP3<br>USP3<br>VIPAS39<br>WWC2<br>XKR4<br>XYLT1<br>ZBTB2<br>ZBTB20<br>ZC3H11A<br>ZCCHC3<br>ZNF367<br>ZNF385A<br>ZNF423<br>ZNF512B<br>ZNF532<br>ZNF652<br>ZNF704<br>ZNRF2<br>ZNRF3
hsa-mir-185-5p	↓	ABCG4<br>ACTN4<br>ANKRD34A<br>ATP6V1F<br>ATP8B1<br>BEND3<br>BICD1<br>BSN<br>CAPZB<br>CBX5<br>CCND2<br>CORO2B<br>CTSK<br>ELOVL4<br>GPR12<br>IGF2R<br>IKZF4<br>KIF3C<br>KLC1<br>LPCAT3<br>LSAMP<br>MAT2A<br>MSI1<br>MYO18A<br>NCOA2<br>PAX2<br>PBX2<br>PCDHA5<br>PITPNA<br>PLA2G2F<br>PRKAR2A

		RERE<br>RIC8B<br>RNF20<br>SAR1A<br>SCARB1<br>SIX3<br>SLC16A2<br>SLC24A2<br>SLC25A15<br>SLC25A53<br>SNX30<br>SORL1<br>SOX13<br>SPATA2<br>TEAD1<br>TMOD2<br>TOX3<br>TRIM44<br>VIPAS39<br>XYLT1<br>ZNF704
hsa-mir-320a-3p	↓	ABHD13<br>ACSS1<br>ACVR2B<br>ADAM10<br>AGFG1<br>AGPS<br>AIF1L<br>AK4<br>AKT3<br>ALKBH5<br>ANKFY1<br>ANKH<br>ANKRD13A<br>ANKRD52<br>AP3M1<br>ARFGEF2<br>ARFIP1<br>ARL5A<br>ARL8B<br>ARMC1<br>ARMCX2<br>ARPP19<br>ATF7IP2<br>ATG14<br>ATL3<br>ATP11A<br>ATRX<br>AZIN1<br>B3GALNT2<br>BANP<br>BLCAP

	BMI1
	BMPR1A
	BOD1L1
	BRWD3
	BVES
	C21orf91
	CACNA1E
	CACNG2
	CALN1
	CAPRIN1
	CCR7
	CD274
	CDH2
	CDH20
	CDK13
	CDK6
	CELSR3
	CFL2
	CHGA
	CHIC1
	CHSY3
	CISD1
	CISD2
	CKAP5
	CLCN5
	CNKSR2
	CNOT7
	CNPY4
	COMMD3-BMI1
	COPS2
	CPD
	CREB3L2
	CREBRF
	CRKL
	CTDNEP1
	CYLD
	CYTH1
	DAB2
	DAG1
	DAZAP1
	DBN1
	DCBLD2
	DCC
	DCP1A
	DDX42
	DENND5B
	DESI2
	DHDDS
	DHX15
	DLGAP4
	DLK1
	DLX1

	DMD
	DNAJA2
	DNAJC3
	DNER
	DOK6
	DPY30
	DR1
	DTNA
	E2F1
	EFS
	EGR3
	EIF3J
	ELL2
	ELOVL6
	EMB
	EMC7
	EMILIN2
	ENAH
	ENPEP
	ENTPD4
	EOGT
	ESRRG
	ETFA
	ETV1
	EXO1
	EXOC5
	EYA3
	FAM117B
	FAM135B
	FAM160B1
	FAM199X
	FANCF
	FBXO28
	FBXO42
	FKBP5
	FLRT3
	FOXM1
	FOXQ1
	FUS
	GABPB2
	GLIS3
	GMCL1
	GNAI1
	GNS
	GOLGA1
	GOLGA3
	GPBP1
	GRIN2A
	GSPT1
	GTPBP2
	GXYLT1
	HECA

	HECTD2
	HELZ
	HERPUD1
	HEY1
	HIC2
	HIVEP2
	HLCS
	HLTF
	HNRNPF
	HOXA10
	HOXB4
	HSPA4
	HSPH1
	HUNK
	IDE
	IGF2BP3
	ING5
	INO80D
	INVS
	IPO5
	IPO7
	IRF6
	KCNAB1
	KCNS3
	KCTD9
	KIN
	KITLG
	KLHDC9
	KLHL14
	KLHL36
	KRI1
	LHFPL3
	LMO3
	LOXL3
	LPIN3
	LRP6
	LRRC4
	MAK16
	MAP1LC3B
	MAP7
	MAPK1
	MAPK8IP3
	MBLAC2
	MED21
	MEX3B
	MIER1
	MIER3
	MITF
	MLLT3
	MMP16
	MN1
	MSANTD4

	MSI2
	MTDH
	MTF2
	MTMR12
	MXI1
	MYEF2
	MYL12A
	MYRF
	N4BP1
	NAA20
	NAA40
	NABP1
	NAP1L5
	NCAPD3
	NCDN
	NECAP2
	NEGR1
	NFIA
	NKX2-4
	NR2C2
	NR3C1
	NRK
	NRN1
	NRP1
	NUFIP2
	NXT2
	ONECUT2
	OTUD7B
	PAN3
	PBX1
	PBX3
	PCDH19
	PCDHA1
	PCDHA10
	PCDHA11
	PCDHA12
	PCDHA13
	PCDHA2
	PCDHA3
	PCDHA4
	PCDHA5
	PCDHA6
	PCDHA7
	PCDHA8
	PCDHA9
	PCDHAC1
	PCDHAC2
	PCGF5
	PCSK1
	PCSK6
	PCSK7
	PDCD4

	PDS5A
	PDZD8
	PFKM
	PHC1
	PHF1
	PI15
	PIK3CA
	PIK3R1
	PLEKHA5
	PLK3
	PLXDC2
	POLR1C
	PPCS
	PPM1H
	PPP1R16B
	PPP1R3E
	PPP2R2C
	PRKAG2
	PRPS1
	PSMF1
	PTEN
	PTPRB
	QSOX2
	RAB11A
	RAB18
	RAB3GAP2
	RAD51
	RAI2
	RANBP2
	RAP1A
	RAPGEFL1
	RASA1
	RASSF8
	RBM24
	RBMS3
	RCN2
	RELT
	RIT1
	RMDN3
	RNF185
	RPL15
	RRP1B
	RUNX1T1
	SASH1
	SATB2
	SCAI
	SCN1A
	SDHD
	SDK1
	SEC63
	SEMA3A
	SERINC3

	SESN3
	SESTD1
	SETD6
	SF3B3
	SGCB
	SH2B3
	SIN3A
	SLC10A3
	SLC10A7
	SLC28A3
	SLC5A3
	SLC6A8
	SMARCC1
	SMARCD2
	SMG7
	SNTB2
	SOBP
	SORCS1
	SOX4
	SPOPL
	SREK1
	SREK1IP1
	SRP19
	ST3GAL1
	STAG2
	STARD4
	STAT4
	STIM2
	STK24
	SYNGR2
	TANC1
	TANC2
	TBC1D15
	TBX18
	TBX4
	TFAP2B
	TFCP2L1
	THSD7A
	THUMPD3
	TIFAB
	TIMM8B
	TIPARP
	TLL2
	TMEM106B
	TMEM108
	TMEM167B
	TMEM255A
	TOR1AIP2
	TPD52
	TPD52L2
	TPM3
	TRABD2B

		TRIAP1
		TSC1
		TSC22D4
		TSHZ3
		TUSC3
		UBE2Z
		ULK1
		UNKL
		USP44
		USP46
		VDAC1
		VLDLR
		VPS36
		VPS37B
		WDR1
		WLS
		WNT9B
		WWC2
		XIAP
		XPO1
		YIPF6
		YOD1
		YWHAH
		ZBTB10
		ZC3H12C
		ZC3H7B
		ZDHHC21
		ZDHHC3
		ZFP91
		ZIC3
		ZNF281
		ZNF516
		ZNF652
		ZSWIM6

Table S2. Summary of the significant molecular KEGG pathways related to cellular processes (A, B, C, D) and diseases (F, G) modulated by the selected miRNAs in CPP.

A) Signal transduction

KEGG pathway	Adjusted p-value	No. of genes
PI3K-Akt signaling pathway	8.31E-023	54
MAPK signaling pathway	1.16E-022	47
Rap1 signaling pathway	1.02E-017	38
Ras signaling pathway	4.28E-015	35
Wnt signaling pathway	5.72E-012	25
Hippo signaling pathway	5.72E-012	26
Calcium signaling pathway	5.13E-010	25
FoxO signaling pathway	1.06E-009	22
HIF-1 signaling pathway	2.15E-009	19

Jak-STAT signaling pathway	1.62E-007	20
ErbB signaling pathway	1.58E-006	14
Hedgehog signaling pathway	3.03E-005	10
mTOR signaling pathway	1.08E-004	10
TGF-beta signaling pathway	1.94E-004	11
Phosphatidylinositol signaling system	1.94E-004	11
TNF signaling pathway	4.71E-004	12
Notch signaling pathway	4.24E-003	7
VEGF signaling pathway	4.80E-002	6

B) Nervous system

KEGG pathway	Adjusted p-value	No. of genes
Neurotrophin signaling pathway	3.05E-011	23
Dopaminergic synapse	7.38E-010	22
Cholinergic synapse	1.48E-009	20
Retrograde endocannabinoid signaling	9.92E-008	17
Long-term potentiation	1.42E-006	13
Glutamatergic synapse	1.52E-006	16
Long-term depression	3.39E-006	12
Serotonergic synapse	3.38E-005	14
GABAergic synapse	3.39E-004	11

C) Substance Dependence

KEGG pathway	Adjusted p-value	No. of genes
Morphine addiction	1.62164E-07	16
Amphetamine addiction	1.62164E-07	14
Cocaine addiction	1.13298E-05	10
Alcoholism	0.000267754	16
Nicotine addiction	0.00935854	6

D) Endocrine System

KEGG pathway	Adjusted p-value	No. of genes
Melanogenesis	3.78E-10	20
Insulin signaling pathway	3.65E-08	20
Thyroid hormone signaling pathway	4.26E-07	17
Estrogen signaling pathway	1.32E-06	15
Prolactin signaling pathway	2.08E-06	13
Insulin secretion	8.38E-06	13
Progesterone-mediated oocyte maturation	1.13E-05	13
GnRH signaling pathway	1.96E-05	13
Adipocytokine signaling pathway	0.000358	10

E) Immune system

KEGG pathway	Adjusted p-value	No. of genes
Fc gamma R-mediated phagocytosis	1.59131E-05	13
T cell receptor signaling pathway	4.56757E-05	13
Leukocyte transendothelial migration	5.85734E-05	14
Fc epsilon RI signaling pathway	0.000267754	10
Chemokine signaling pathway	0.000358264	16
B cell receptor signaling pathway	0.000358264	10
RIG-I-like receptor signaling pathway	0.0301527	7

F) Cancer

KEGG pathway	Adjusted p-value	No. of genes
Pathways in cancer	1.63825E-25	56
Transcriptional misregulation in cancer	5.34E-019	37
Proteoglycans in cancer	2.07E-017	38
MicroRNAs in cancer	4.29E-014	39
Melanoma	1.62E-011	19
Chronic myeloid leukemia	2.37E-011	19
Prostate cancer	7.56E-011	20
Pancreatic cancer	2.85E-010	17
Glioma	3.50E-009	16
Small cell lung cancer	1.08E-008	17
Colorectal cancer	5.11E-008	14
Bladder cancer	4.26E-007	11
Non-small cell lung cancer	1.62E-006	12
Renal carcinoma	2.57E-006	12
Viral carcinogenesis	7.62E-006	20
Acute myeloid leukemia	7.19E-005	10
Basal cell carcinoma	1.94E-004	9
Thyroid cancer	2.68E-004	8
Endometrial cancer	7.47E-004	7

G) Viral Infectious disease

KEGG pathway	Adjusted p-value	No. of genes
HTLV-1 infection	5.30E-015	**37**
Hepatitis B	6.10E-010	23
Amoebiasis	1.61E-007	17
Chagas disease (American trypanosomiasis)	3.05E-006	15
Epstein-Barr virus infection	5.71E-006	20
Measles	1.62E-004	14
Influenza A	1.94E-004	16
Toxoplasmosis	7.98E-004	12
Herpes simplex infection	3.35E-003	14
Hepatitis C	9.11E-003	11

Table S3. Summary of the significant molecular KEGG pathways related to cellular processes (A, B, C) and diseases (D, E) modulated by the selected miRNAs in CSP.

A) Signal transduction

KEGG pathway	Adjusted p-value	No. of genes
ErbB signaling pathway	3.65E-05	9
mTOR signaling pathway	3.65E-05	8
Ras signaling pathway	0.000139122	12
MAPK signaling pathway	0.000448852	12
PI3K-Akt signaling pathway	0.000448852	14
Rap1 signaling pathway	0.000448852	11
FoxO signaling pathway	0.000459	9
VEGF signaling pathway	0.001262	6
Hippo signaling pathway	0.003356	8
TNF signaling pathway	0.003411	7
Calcium signaling pathway	0.007831	8
Phosphatidylinositol signaling system	0.02914	5
Wnt signaling pathway	0.0431235	6

B) Endocrine system

KEGG pathway	Adjusted p-value	No. of genes
Melanogenesis	4.49E-004	8
Insulin signaling pathway	4.84E-004	9
Thyroid hormone signaling pathway	9.48E-004	8
Estrogen signaling pathway	1.75E-003	7
Prolactin signaling pathway	2.79E-003	6
Progesterone-mediated oocyte maturation	6.05E-003	6

C) Immune system

KEGG pathway	Adjusted p-value	No. of genes
T cell receptor signaling pathway	4.49E-004	8
Toll-like receptor signaling pathway	5.26E-004	8
Leukocyte transendothelial migration	1.14E-003	8
Fc gamma R-mediated phagocytosis	1.26E-003	7
Chemokine signaling pathway	2.90E-003	9
Fc epsilon RI signaling pathway	1.56E-002	5
B cell receptor signaling pathway	1.83E-002	5

D) Viral infectious disease

KEGG pathway	Adjusted p-value	No. of genes
Hepatitis B	3.65035E-05	11

HTLV-1 infection	0.000113102	13
Chagas disease (American trypanosomiasis)	0.000113102	9
Toxoplasmosis	0.000212993	9
Influenza A	0.000477164	10
Hepatitis C	0.00205935	8
Epstein-Barr virus infection	0.00432372	9
Measles	0.00845821	7

E) Cancer

**KEGG pathway**	**Adjusted p-value**	**No. of genes**
Pathway in cancer	2.22973E-09	22
Transcriptional misregulation in cancer	9.45E-006	13
Small cell lung cancer	3.65E-005	9
Renal cell carcinoma	3.65E-005	8
Pancreatic cancer	4.24E-005	8
Glioma	5.49E-005	8
Melanoma	7.04E-005	8
MicroRNAs in cancer	1.39E-004	14
Proteoglycans in cancer	1.39E-004	12
Non-small cell lung cancer	1.60E-004	7
Chronic myeloid leukemia	4.59E-004	7
Viral carcinogenesis	1.26E-003	10
Prostate cancer	1.26E-003	7
Colorectal cancer	1.26E-003	6
Endometrial cancer	4.02E-003	5
Acute myeloid leukemia	7.83E-003	5
